# 2124. Novel Polygalacturonic and Caprylic Acid (PG+CAP) Antimicrobial Wound Ointment Effectiveness in Eradicating *C. auris* Biofilms In Vitro

**DOI:** 10.1093/ofid/ofad500.1747

**Published:** 2023-11-27

**Authors:** Y Lan Truong, Bahgat Z Gerges, Joel Rosenblatt, Issam I Raad

**Affiliations:** UT MD Anderson Cancer Center, Houston, Texas; MD Anderson UT, Missouri City, Texas; MD Anderson UT, Missouri City, Texas; MD Anderson UT, Missouri City, Texas

## Abstract

**Background:**

*Candida auris* (CA) is a multidrug-resistant fungal pathogen of increasing concern and has caused infection outbreaks in hospitals as a result of nosocomial transmission. CA has caused both invasive bloodstream and wound infections with significant morbidity and mortality. Recently, a novel polygalacturonic and caprylic acid (PG+CAP) antimicrobial wound ointment was highly effective in healing microbially contaminated chronic wounds in a pilot prospective randomized clinical trial when compared to MediHoney. In order to better combat CA wound infections, here we assessed the effectiveness of PG+CAP and MediHoney wound ointments in eradicating mature CA biofilms in vitro.

**Methods:**

A well-established biofilm eradication model was used to quantify antimicrobial efficacy. Forty-eight-hour biofilm of two different strains of *CA* (AR0381 and AR0382) were formed separately on silicone discs and then exposed to PG+CAP or MediHoney wound ointment for two hours. The silicone discs were sonicated in neutralizer, then serially diluted, plated and counted to enumerate viable colonies remaining following exposure to the ointments. Positive controls were discs that had no wound ointment treatment.

**Results:**

Figure 1 presents the median number of viable colonies recovered from 6 replicates following two-hour exposures of the mature CA biofilms to the wound ointments and positive control.

**Figure:**
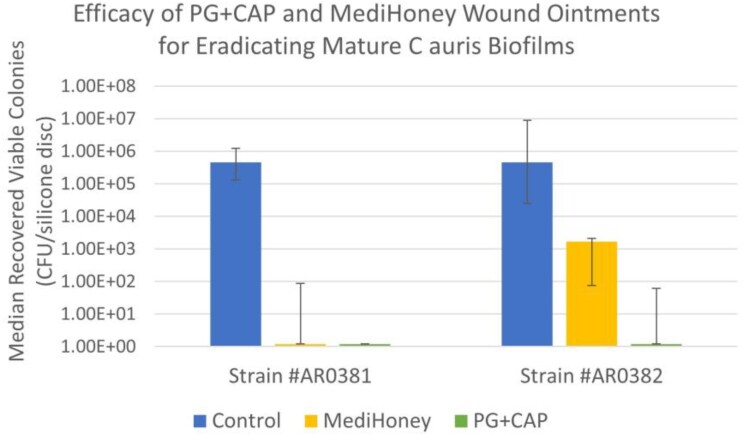

**Conclusion:**

Both ointments fully eradicated biofilm for CA strain AR0381. PG+CAP fully eradicated AR0382 biofilm (greater than 5-log reduction) but MediHoney achieved less than a 3-log reduction against AR0382. The difference between PG+CAP and MediHoney for AR0382 was significant (p = 0.02). These results suggest PG+CAP might be a promising ointment for use in treating CA wound infections and merits further study.

**Disclosures:**

**Joel Rosenblatt, PhD**, Novel Anti-Infective Technologies, LLC: Licensed Technology **Issam I. Raad, Distinguished Professor**, Novel Anti-Infective Technologies, LLC: Technology License

